# Identifying potential drug targets in hepatocellular carcinoma based on network analysis and one-class support vector machine

**DOI:** 10.1038/s41598-019-46540-x

**Published:** 2019-07-18

**Authors:** Zhan Tong, Yuan Zhou, Juan Wang

**Affiliations:** Department of Biomedical Informatics, School of Basic Medical Sciences, Peking University, Beijing, 100191 China

**Keywords:** Machine learning, Target identification

## Abstract

Hepatocellular carcinoma (HCC) is one major cause of cancer-related death worldwide. But now, the systematic therapy for the advanced stages of HCC is rather limited. Thus, the discovery of novel drug targets and thereafter targeted drugs against HCC is continuously needed. In this study, we combined clinical association data, gene expression profiles and manually collected drug target genes with the human protein-protein interaction (PPI) network to establish an *in-silico* HCC drug target predictor. First, we found drug target genes (DTGs), disease-associated genes (DAGs), prognostic unfavorable genes (PUGs) and cancer up-regulated genes (URGs) have higher degree, betweenness, closeness centrality, while cancer down-regulated genes (DRGs), prognostic favorable genes (PFGs) have lower degrees, in comparison with background genes. Moreover, DTG nodes were shown to be closer to DAG, PUG and URG nodes, but farther away from PFG and DRG nodes. Compared to the background, PFGs and DRGs were shown to have relatively bigger genetic dependency scores, while PUGs and URGs have smaller genetic dependency scores. Finally, based on the observed features of DTGs, we constructed a drug target predictor using one-class support vector machine (one-class SVM). Performance evaluation results suggested our predictor could effectively identify putative drug target genes for further research.

## Introduction

With approximately 800, 000 deaths each year, primary liver cancer is one of the leading causes of cancer-related death worldwide, and its incidence is still increasing^1,2^. Hepatocellular carcinoma (HCC), which accounts for approximately 90% of all cases of primary liver cancers, can be caused by a variety of risk factors including hepatitis B virus or hepatitis C virus infection, alcohol abuse, metabolic syndrome and non-alcoholic steatohepatitis^3,4^. Despite the practice for surveillance programmes, HCC is still most frequently diagnosed at advanced stages when potentially curative treatments (i.e. surgical resection, liver transplantation and local ablation) become limited^5^. Sorafenib, which extended the median overall survival of patients with advanced HCC from 8 to 11 months, was the sole systemic therapy approved for the treatment of the advance stages of HCC until 2016^4^. Although new drugs (i.e. lenvatinib, regorafenib, cabozantinib and ramucirumab) have been illustrated to improve clinical outcomes of patients with advanced HCC, the survival improvements are still modest^4,6^. Thus, the development of more effective drugs is still in urgent demands.

In drug discovery, therapeutic failures at late stages of development such as phase II and phase III clinical trials are extremely costly^7^. It has been reported that most failures are caused by drug efficacy lacking or safety issues^8^, which, in turn, are partly due to the lack of a systematic understanding of diseases. Network pharmacology, firstly proposed by Andrew L Hopkins^9^, has been widely accepted as useful tools to evaluate and demonstrate the rationality of drug in a systematic manner. It has been reported that HCC has highly therapy resistance because of its highly genetic heterogeneity^2,10^. And the mechanism underlying HCC pathogenesis is implicated in the alteration of various biological pathways, therefore, drug target identification for HCC based on network pharmacology has its unique advantage. With the development of high-throughput techniques, the quantity and quality of biological interaction data are improved dramatically. Thus, biological network models, including protein-protein interaction (PPI), signaling, transcriptional regulatory and co-expression networks, can be taken as scaffold for investigating alteration and associations of genes in specific disease conditions in a systematic manner^11^. Indeed, previous studies have shown that biological network-based approach is a promising way to detect potential biomarkers and drug targets from multidimensional omics data in diverse diseases^12–15^. However, most previous methods are based only on network topological characteristics and ignore other gene functional features, which may inform its importance in the progression of cancer and patient’s survival. For example, the genetic dependency score, which estimates the importance of one gene for the survival and proliferation of cancer cells by analyzing the genome-scale loss-of-function screen data performed in various human cancer cell-lines, could facilitate the prioritization of cancer-related therapeutic targets^16,17^.

It has been found that drug targets have distinctive topological and biological characteristics in different types of cellular networks^15,18,19^. And machine learning, as a specialized branch of statistics and computer science, is now extensively used in the field of drug discovery^20–22^. In this study, we first mapped different types of genes that potentially related to HCC, to the human PPI network and the human cellular signaling network. Then, statistical analyses of genetic dependency scores and network characteristics were performed in both networks. Next, we developed a novel drug target gene prediction method based on the manually curated drug target genes in HCC. Through integrating genetic dependency scores with network characteristics, we constructed the drug target predictor in HCC based on one-class support vector machine (one-class SVM). Finally, the performance evaluations were conducted through statistical metrics and comparing with the independent dataset.

## Results And Discussion

### Network and genetic dependency score characteristics for different types of genes potentially related to hepatocellular carcinoma

**Figure 1:**
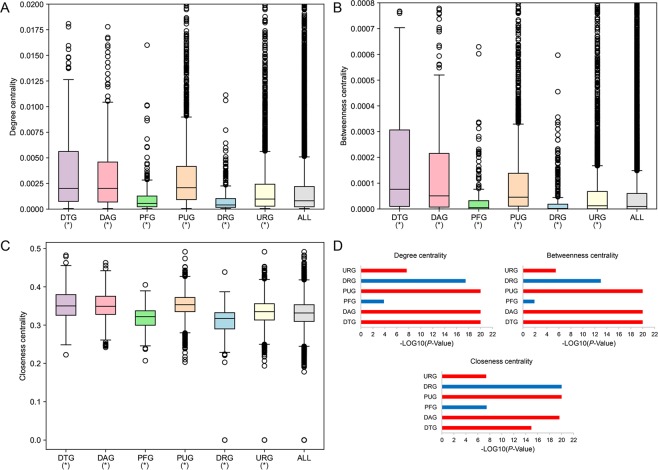
Network centrality characteristics of different types of genes in human PPI network. (**A**) Degree centrality. (**B**) Betweenness centrality. (**C**) Closeness centrality. *P < 0.05 from Wilcoxon test. (**D**) P-values of comparing centralities of DTGs and different types of PDRGs with those of background. Red and blue bars represent the greater and less centralities compared to the background, respectively.

**Figure 2:**
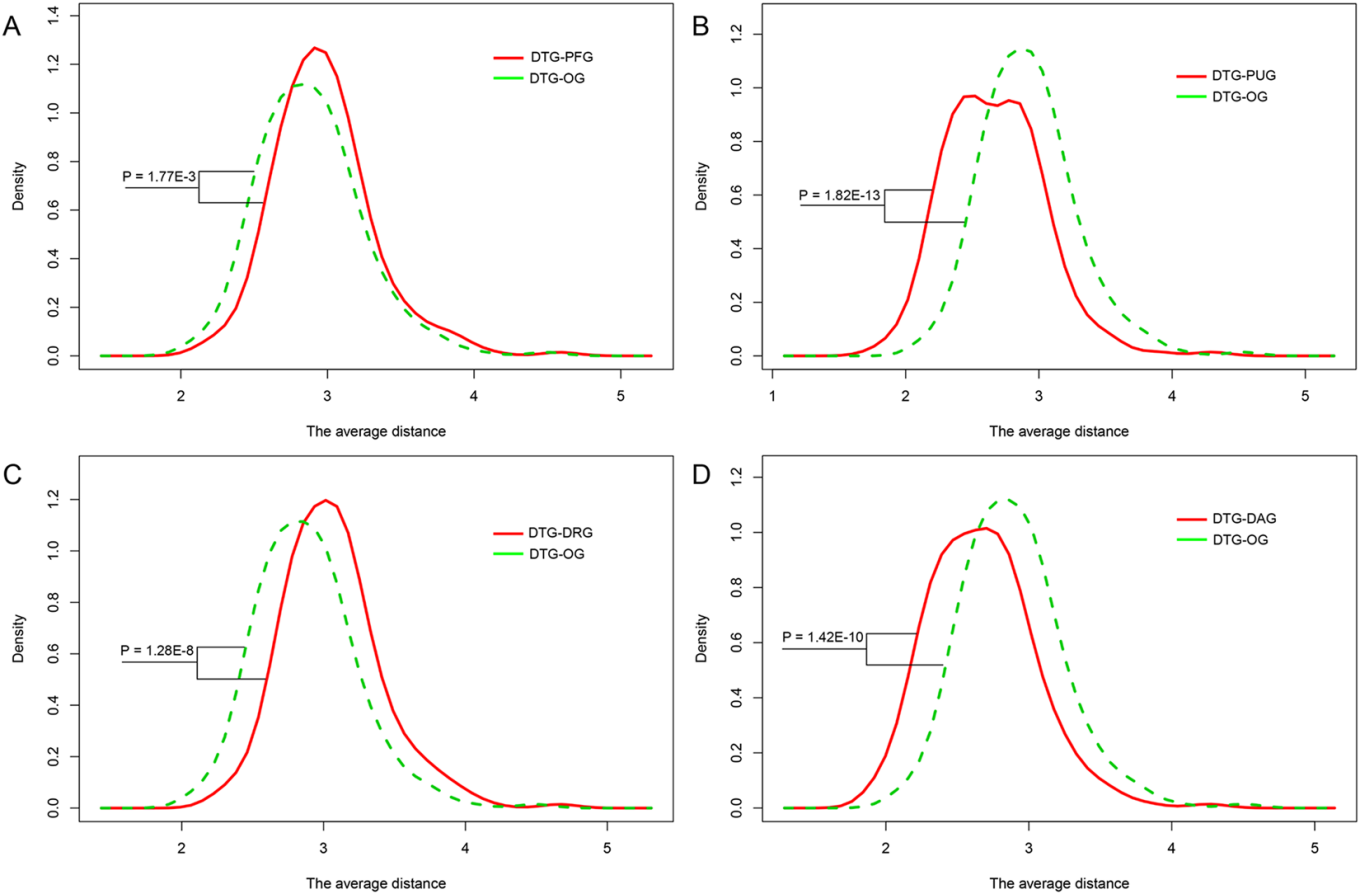
Network distance characteristics between DTGs and the specific PDRGs in the PPI network. Density plots display the probability distribution of the average lengths of shortest paths between groups (DTG-PDRG/DTG-other genes).

Since no genes are isolated but interact with each other^23^, it is a reasonable way to understand the functions of genes in the specific biological networks. To characterize the network topological features of different types of gene nodes, we first investigated the network centrality characteristics of drug target genes (DTGs) and five types of potential disease related genes (PDRGs) in human PPI network, in which 217 DTGs, together with five types of PDRGs including 322 disease-associated genes (DAGs), 230 prognostic favorable genes (PFGs), 2,574 prognostic unfavorable genes (PUGs), 434 cancer down-regulated genes (DRGs) and 4,004 cancer up-regulated genes (URGs) were mapped. Three types of network centralities were considered, including degree centrality, betweenness centrality and closeness centrality. These centrality indices could reflect the connectivity of nodes in biological networks. When compared to all gene nodes in the human PPI network, DTG, DAG, PUG and URG nodes have higher degree centrality, betweenness centrality and closeness centrality, while PFG and DRG nodes have lower centralities (Fig. 1). And among DTG and the five types of PDRG nodes, DTG have the highest degree centrality, betweenness centrality and closeness centrality. To explore how closely the DTG nodes are linked to the PDRG nodes, we estimated the average and minimum lengths of the shortest paths between DTG and PDRG nodes (DTG-PDRG), and with the respective other gene nodes (DTG-OG). Interestingly, DTG nodes are closer to DAG and PUG nodes, but farther away from PFG and DRG nodes, in comparison with the respective OG nodes (Fig. 2), indicating the non-random distribution of DTGs and PDRGs in human PPI network.

The observed network characteristics were further validated in the human cellular signaling network, in which 184 DTGs, 236 DAGs, 106 PFGs, 1,053 PUGs, 225 DRGs and 1,418 URGs were mapped. And since the links of human cellular signaling network are directional, additional analyses of in-degree and out-degree centralities were also performed. The results are largely similar to those in the PPI network: when compared to all gene nodes in the signaling network, DTG, DAG and PUG nodes have higher degree, betweenness, closeness, in-degree and out-degree centralities (Supplementary Fig. S1). The only differences are that DRGs have higher betweenness and closeness centralities, and no significant centrality differences were detected for URGs. In distance analysis, when compared to the respective OG nodes, DTG nodes are closer to all types of PDRG nodes except URG nodes (Supplementary Fig. S2), indicating the DTGs and PDRGs are clustered together in the signaling network.

**Figure 3:**
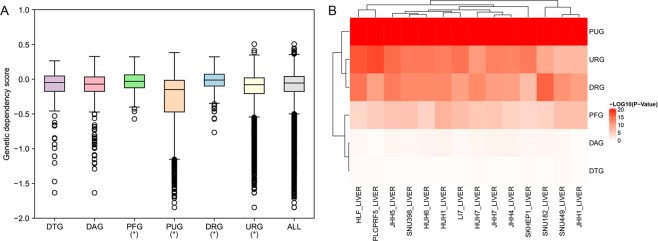
Genetic dependency score characteristics of different types of genes. (**A**) Box plot displays the genetic dependency score differences among different gene groups and all genes having genetic dependency scores. *P < 0.05 from Wilcoxon test. (**B**) Heat-map displays the p-values of comparing genetic dependency scores of each gene group with all genetic dependency scores with Wilcoxon test in 14 HCC cell-lines.

Although the network centrality could partly reflect the importance of a gene, the genetic screen is still the golden standard to evaluate gene importance. The genetic dependency scores which integrated the genetic screen results among 14 liver cancer cell-lines were employed to analyze the gene importance characteristics of the DTGs and PDRGs. Interestingly, PFGs and DRGs were shown to have relatively bigger genetic dependency scores (indicating lower gene importance), while PUGs and URGs have smaller genetic dependency scores (indicating higher gene importance), in comparison with all genes whose genetic dependency scores are available (Fig. 3A,B). However, no statistical significance is found for DTGs and DAGs. To have a better view of genetic dependency scores for the neighboring region of each gene, we computed the average values of genetic dependency scores among the neighbor nodes of each gene in the human PPI network and the signaling network, respectively. When compared to other genes’ neighbors, DRG neighbors were shown to have bigger genetic dependency scores, while PUG and URG neighbors have smaller genetic dependency scores (Supplementary Fig. S3). We also found DAG neighbors have significant smaller genetic dependency scores in both networks. These results indicate the gene importance of DTGs, PDRGs and their network neighbors are also diverged from other background genes in the networks.

### Drug target prediction and performance evaluation

**Figure 4:**
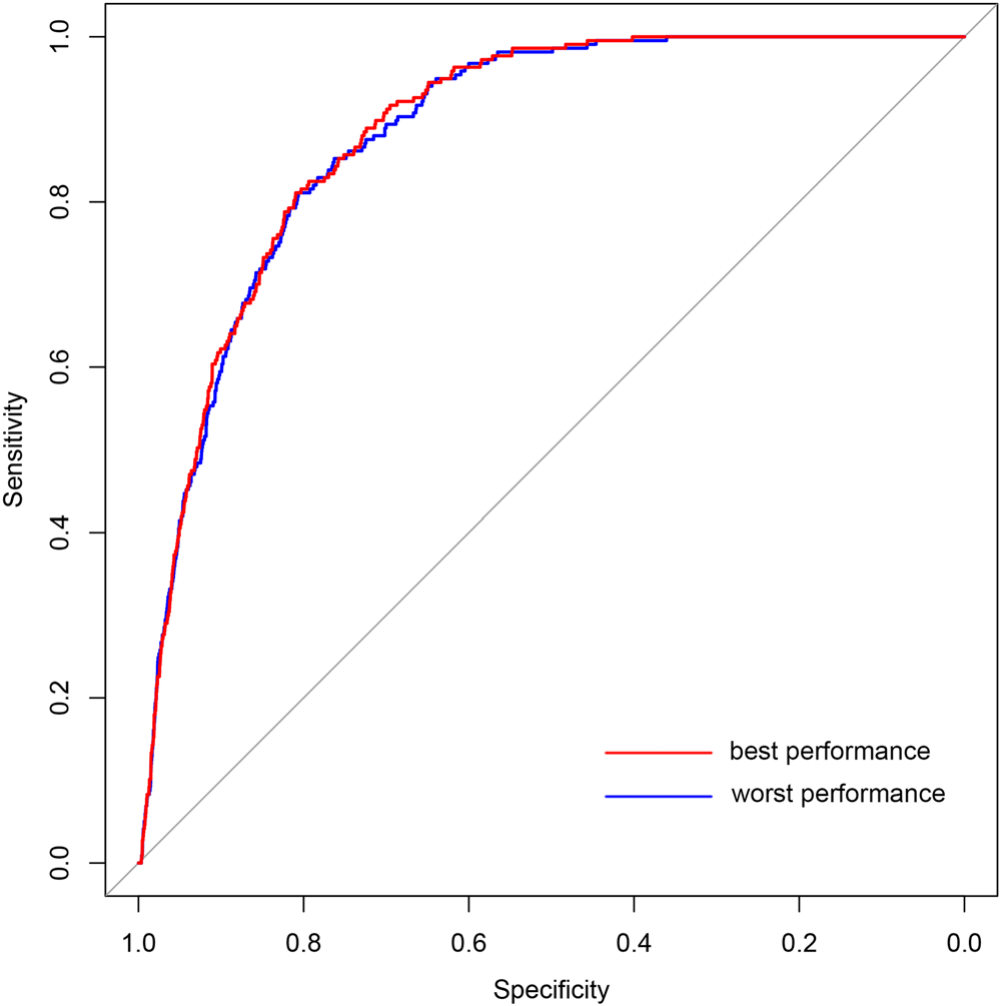
ROC curves of drug target predictions. Curve in red represents the best performance in ten rounds of predictions (AUC = 0.8849), while curve in blue represents the worst (AUC = 0.8820).

From the above statistical analysis, we found DTGs have more significant network characteristics in the human PPI network in comparison with the human signaling network. Thus, we constructed our drug target predictor by integrating genetic dependency scores with network features obtained from the human PPI network. A total of 217 anti-HCC drug target genes (DTGs) mapped to the human PPI network were considered as the positive samples, while 17,040 genes apart from these DTGs in the human PPI network were considered as the negative samples. We noted that the actual distinction between the positive and negative samples is elusive, since many collected anti-HCC drugs are still under clinical trials and there should be many not yet discovered drug targets in the negative samples on the other hand. We therefore used one-class SVM to build our predictor. One-class SVM aims at depicting the region defining each class of samples rather than identifying a boundary discriminating two classes^20^, and is generally more suitable for the drug target prediction task since it is more robust to the potentially false negative samples. We first evaluated the performance of our one-class SVM predictor by five-fold cross-validation. To reduce the influence of random partition of training and testing subsets in cross-validation tests, the five-fold cross-validation was repeated ten times with different partitions of training and testing subsets. The prediction performance is summarized in Table 1, where the average of the AUC scores of ten rounds of evaluation tests achieved 0.8834, with the worse result as 0.8820. This result suggests that our drug target predictor has relatively robust prediction performance (Fig. 4).

**Table 1:** Performance summary of ten repeats of five-fold cross-validation tests.

Sensitivity	Specificity	MCC	AUC
61.29%	91.16%	0.1993	0.8832
64.52%	88.76%	0.1833	0.8820
58.53%	91.57%	0.1944	0.8837
58.99%	90.80%	0.1863	0.8827
53.00%	92.79%	0.1905	0.8845
49.31%	93.63%	0.1887	0.8825
60.37%	91.07%	0.1948	0.8849
58.99%	90.72%	0.1854	0.8834
65.44%	89.10%	0.1898	0.8827
62.21%	89.96%	0.1882	0.8843

One critical issue is whether the selection of network centrality measures as the input features could result in bias and over-estimation of prediction performance, because disease-related genes often show higher network centralities than other genes in the genome background. To this end, we re-trained the one-class SVM model after excluding all network centrality features. The cross-validation results indicate there is no significant change in AUC scores, with the average AUC score of 0.8838 (Supplementary Table S1). This result indicates that the network centrality features are not the most informative features in our model and do not introduce prominent bias to our prediction model. We further checked whether selection of particular network centrality features would have a considerable impact on the performance. Three additional centrality measures, i.e. PageRank centrality, eigenvector centrality and Katz centrality, were included into the feature set to re-train the one-class SVM model. We found integrating additional network centrality features have no significant impact on the performance, with the average AUC scores of 0.8838 (see Supplementary Table S2). In fact, we found all of the additional network centrality measures significantly correlated with one of previous centrality measures (i.e. closeness centrality with eigenvector centrality and Katz centrality, degree centrality with PageRank centrality), with spearman correlation coefficient above 0.97 (Supplementary Fig. S4). Furthermore, one network-based disease gene prediction algorithm, DADA proposed several statistical adjustment strategies to combine existing approaches to reduce the degree bias of disease genes^24^. So we also tried to use the prediction scores of DADA algorithm as the alternative network features, and assessed the prediction performance using the same ten repeats of five-fold cross-validation. Better performance of our primary feature set than DADA scores was observed (average AUC, 0.8569 vs 0.8162, Supplementary Table S3), while replacing network centrality features with DADA scores, or appending the DADA score to the primary feature set do not significantly alter the prediction performance (average AUC, 0.8558 and 0.8562, respectively, Supplementary Table S3). These results suggest that the network centrality bias does not result in significant overestimation of the prediction performance for our one-class SVM model. Therefore, we kept the primary network centrality feature selection for the subsequent analysis. To make our prediction results more stable, the average scores of ten rounds of evaluation tests were taken as the final prediction scores, and only the genes which met all the cut-offs of ten tests were taken as the putative drug target genes in HCC. Finally, 1,143 genes were predicted as the putative drug target genes, which composite 6.62% of the coding genes in human genome.

**Figure 5:**
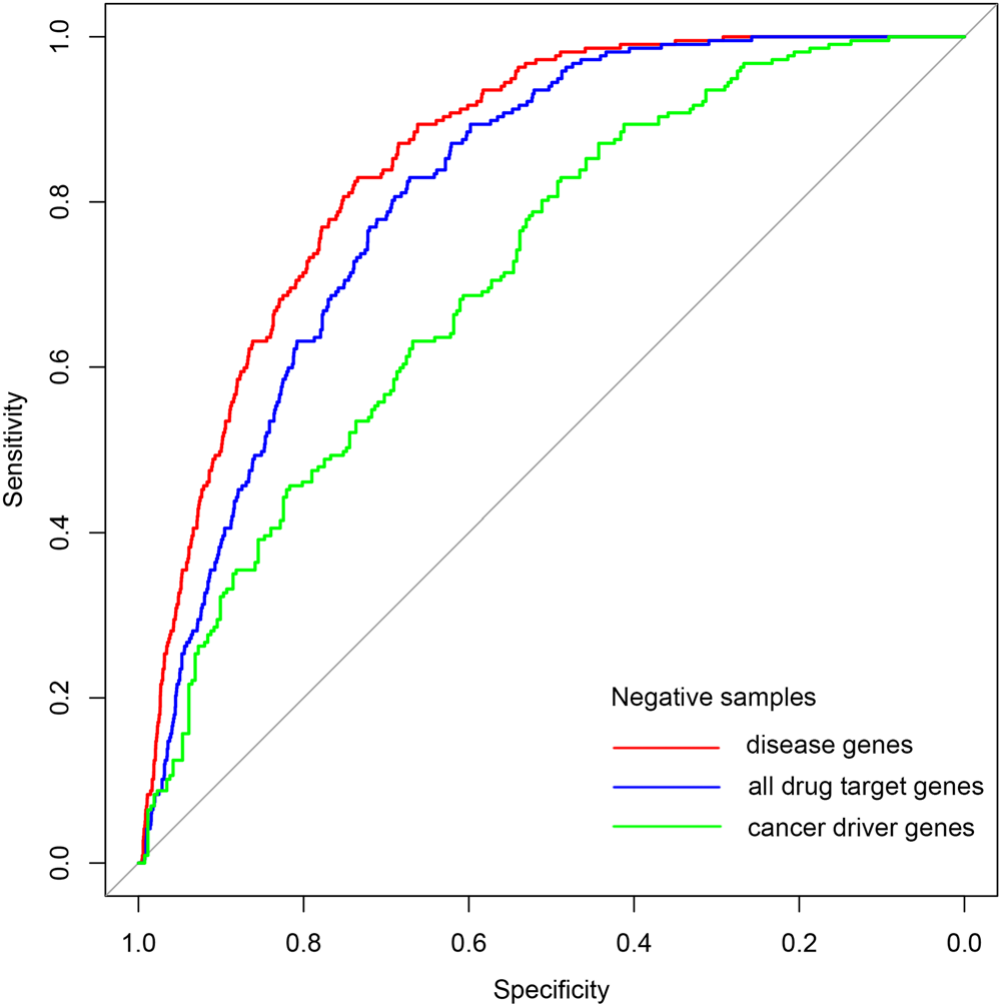
ROC curves of combining the final prediction scores with different types of disease related genes as negative sample sets. Curve in red represents the performance using curated disease genes from DisGeNET database as negative samples (AUC = 0.8536). Curve in blue represents the performance using known human drug target genes from DrugBank database as negative samples (AUC = 0.8122). And curve in green represents the performance using cancer driver genes as negative samples (AUC = 0.7132).

We further evaluate our predictor using an independent criterion of positive-negative partition, i.e. to check if our predicted HCC drug targets are enriched for known targets of anti-cancer drugs. To test this hypothesis, we first obtained the anti-cancer drug target genes by parsing associated files downloaded from DrugBank database. Then, we calculated the proportions of anti-cancer drug target genes in the putative drug target gene set and the background gene set. We found the proportion of anti-cancer drug target genes in the predicted drug target gene set is significant higher than that in the background gene set (24.51% versus 9.35%, P-value = 2.20E-46 by Fisher’s exact test). This result confirms that our method could effectively identify anti-cancer drug target genes against HCC. Take in consideration many studies have illustrated that drug target and other kinds of disease-relevant genes have specific network properties, it is also interesting to explore whether our method could distinguish anti-HCC drug target genes from various types of disease-relevant genes. So we used the final prediction scores and collected three different types of disease-relevant genes as our negative sample sets respectively (including curated disease genes from DisGeNET database, known human drug target genes from DrugBank database and cancer driver genes from Matthew H. Bailey *et al*.’s study^25^) to re-estimate the performance of our model. As depicted by the ROC curves in Fig. 5, our method could effectively distinguish anti-HCC drug target genes from other known disease genes and drug target genes with considerable performance. Although the distinction between cancer driver genes and anti-cancer drug targets is much more challenging since cancer driver genes could often serve as the anti-cancer drug targets, our model could still distinguish anti-HCC drug target genes from the known cancer driver genes with moderate prediction accuracy (Fig. 5), indicating the robustness of our prediction model.

**Figure 6:**
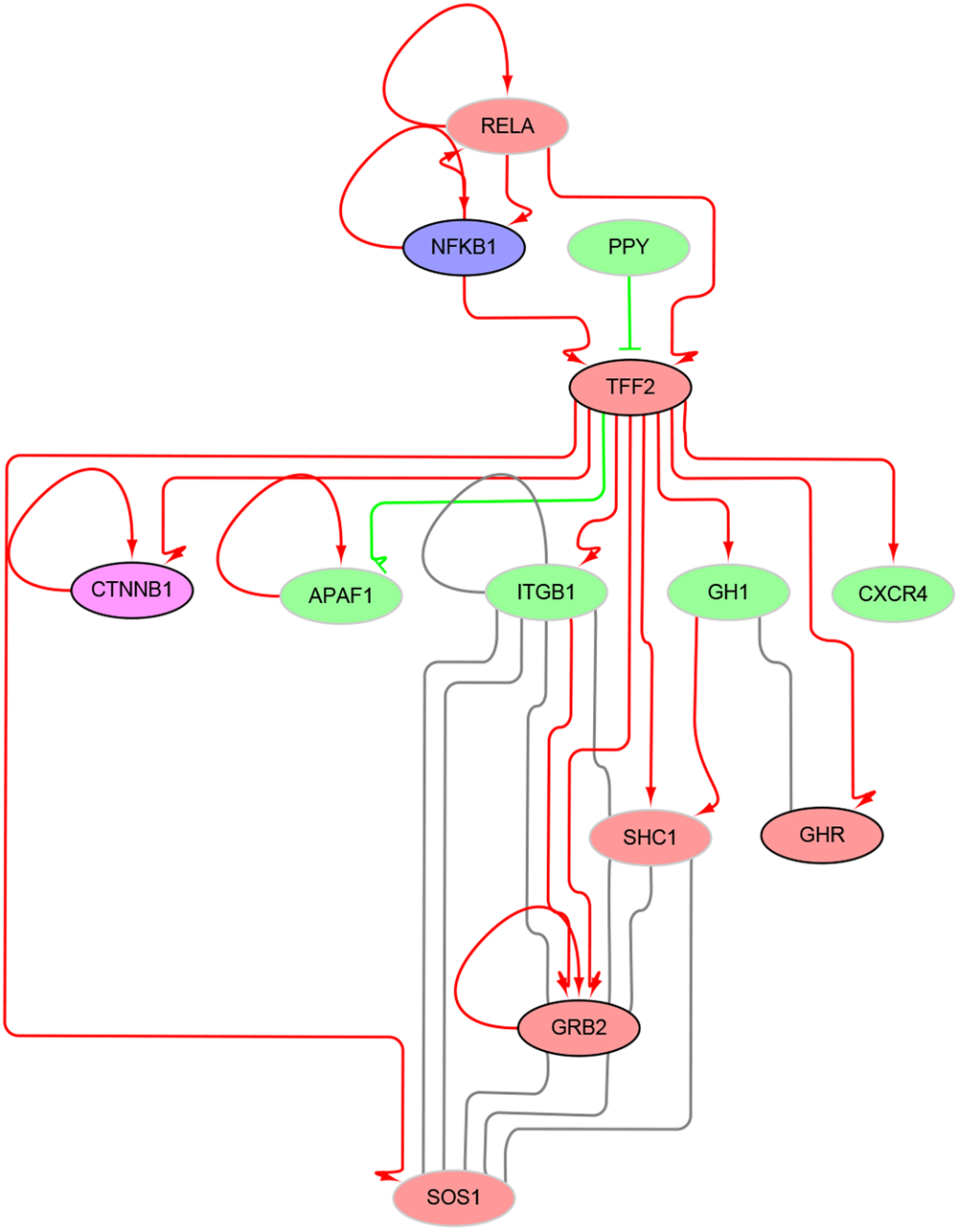
Example view of putative drug target gene in the signaling network. Nodes in blue, purple, red and green represent DTG, DTG as well as PDRG, PDRG and other gene nodes, respectively. Predicted positive gene nodes are with black border, and predicted negative nodes are with gray border. Edges in arrow (red), T-shaped (green) and full line (gray) represent positive, negative and physical interactions, respectively.

To exemplifying the possible functional mechanisms of potential drug targets, here we obtained the sub-network constituted by TFF2 (trefoil factor 2) and its neighbors in the signaling network (Fig. 6). With this view, we could intuitively find TFF2 is a pathology unfavorable gene and more than half of its neighbors are HCC-related. Interestingly, we found there occur known drug targets in both upstream and downstream signaling of TFF2 (NFKB1 and CTNNB1, respectively). Similar to TFF2, 1,143 genes were predicted as the putative drug target genes against HCC, and the full list is available as Supplementary Dataset 1. We hope our prediction results could play an important role in the research of pathogenesis of HCC, and provide useful clues for the design of novel anti-HCC drugs. In the meanwhile, this study could also serve as a sample for the application of network pharmacology in other complex diseases. Nevertheless, there are some apparent limitations in this study. First, through integrating more biological information, such as gene ontology annotations (i.e. molecular function, cellular component and biological process) and druggable information, additional filtering restrictions should be added to obtain more sophisticated prediction results. And taking in consideration of the multi-target therapy for complex diseases, the prediction of drug target combinations should be enabled in the following study. In all, we hope the proposal of more advanced computational methods could facilitate the researches in the field of drug discovery for complex diseases in the future.

## Methods

### Collection of drug targets, disease associated genes and prognostic association genes in HCC

We first searched DrugBank database (https://www.drugbank.ca/) with the keywords ‘Hepatic cancer metastatic’, ‘Relapsed Hepatocellular carcinoma’, ‘Unresectable Hepatocellular Carcinoma’ and ‘Refractory Hepatocellular Carcinoma’^26^. Next, we manually collected target genes of approved drugs in these diseases (13 in total). To expand our positive datasets, we also collected target genes of the drugs used in the clinical trials of the disease ‘Hepatocellular, Carcinoma’. Finally, we got a total of 222 drug target genes (DTGs) in HCC. And all the target genes were standardized as gene official symbol.

We also retrieved disease-associated genes (DAGs) of hepatocellular carcinoma with the keywords ‘Liver neoplasm’ and ‘Liver Carcinoma’ from DisGeNET database (http://www.disgenet.org/, only curated gene-disease associations were considered)^27^. All HCC prognostic association genes with statistical significance were obtained from Human Protein Atlas database (http://www.proteinatlas.org/) with the keyword ‘liver cancer’^28^. Based on whether the gene is prognostic favorable or not, we further classified hepatocellular carcinoma prognostic genes into prognostic favorable genes (PFGs) and prognostic unfavorable genes (PUGs).

### Identification of differentially expressed genes (DEGs) in hepatocellular carcinoma

We first downloaded the RNA-seq data of the ‘TCGA-LIHC’ project from the TCGA database (https://portal.gdc.cancer.gov/). Based on clinical annotations provided, we discarded unpaired RNA-seq data (i.e. without non-tumor controls) and those came from patients who had gotten other types of cancers before or were not representative of a hepatocellular carcinoma case. Then, we got 43 pairs of HCC tumor and para-cancerous tissues’ expression data. To gain genes differentially expressed between the tumors and para-cancerous tissue controls, each gene was evaluated by paired t- test and fold change, and the P-values were corrected by false discovery rate (FDR). The following criteria were finally used: FDR ≤ 0.05 and absolute fold change ≥ 2.0. Then according to the values of fold changes, the differentially expressed genes were further classified into up-regulated genes (URGs) and down-regulated genes (DRGs).

### Network analysis and statistical characteristics of genetic dependency scores

The human PPI network was downloaded from the BioGRID database (https://thebiogrid.org/, build 3.4.161) with the deletion of links with proteins from non-human species^29^. The human cellular signaling network was obtained from the previous study (http://www.cancer-systemsbiology.org/)^30^. The Wilcoxon rank sum test was performed to investigate the degree centrality, betweenness centrality and closeness centrality characteristics for the DTG nodes, and the potentially disease-related genes (PDRGs) in the PPI network and the signaling network, respectively. Five types of PDRGs were considered, including disease-associated genes (DAGs), prognostic favorable genes (PFGs), prognostic unfavorable genes (PUGs), up-regulated genes (URGs) and down-regulated genes (DRGs). In the signaling network, Wilcoxon test was also performed to investigate in-degree centrality and out-degree centrality differences for those six types of nodes. As for network distance analysis, we investigated the characteristics of shortest path lengths between DTG nodes and other five types of PDRG nodes, respectively. More specifically, the average and minimum lengths of the shortest paths between one DTG and the PDRGs, and those between one DTG and the other nodes were also compared by Wilcoxon test. All the above centrality and distance calculation were carried out by using the python package NetworkX^31^. The directional information was considered when the shortest path lengths were calculated in the signaling network. Network visualization in signaling network were conducted by Cytoscape^32^.

The Avana CRISPR screen dataset which reflected genetic dependency of a large amount of genes in various cancer cell-lines, was downloaded from Cancer Dependency Map database (https://www.broadinstitute.org/cancer/cancer-dependency-map, build portal-Avana-2018-06-21)^16^. After filtering by cell-lines, we got genetic dependency score data for 14 distinctive liver cancer cell-lines. Wilcoxon test was performed to investigate the genetic dependency score differences for the DTGs, and the five types of PDRGs for different liver cancer cell-lines. In order to investigate the genetic dependency score differences for the neighbors of the above mentioned six types of nodes in the networks, we computed the average genetic dependency scores for the neighbors of the above six types of nodes and compared with the background using Wilcoxon test.

### Construction of drug target predictor with one-class SVM

The one-class support vector machine (one-class SVM) was utilized to predict drug targets against HCC. LIBSVM 3.23 was used to construct one-class SVM models with the radial basis function (RBF) kernel^33^. The parameters for one-class SVM model was optimized by five-fold cross-validation which was repeated for ten times to obtain the stable results. All the manually curated DTGs, which were mapped to the PPI network, were considered as positive samples here. The remaining gene nodes in the PPI network were taken as negative samples. Both network features and genetic dependency scores were used as the inputs to the one-class SVM prediction model. To encode network features for one-class SVM predictor, we incorporated three types of metrics derived from the previous statistical analyses, including network centralities, network distances and network neighbor properties. More specifically, we first calculated degree centrality, betweenness centrality and closeness centrality for each gene node. Additional centrality measures including PageRank centrality, eigenvector centrality and Katz centrality, were also computed for each gene node to evaluate the impact of the selection of centrality features on prediction performance of one-class SVM model. The distances between each node and seven types of gene nodes (including DTGs, five types of PDRGs and other genes) were also calculated. In addition, the ratios of seven types of gene nodes among one gene’s network neighbors were also computed. Finally, the average genetic dependency scores across 14 distinctive liver cancer cell-lines were obtained for each gene and its network neighbors. Integration of the above features result in a feature vector with 52 feature values for each gene, including 3 network centrality features, 14 network distance features, 7 network neighborhood features, 14 genetic dependency score features of the gene and 14 genetic dependency score features of its network neighbors (See Supplementary Table S4 for details).

To compare the prediction performance of our method with the DADA algorithm, we first constructed DADA dataset by intersecting our PPI network nodes with the network genes covered by the software suite of DADA algorithm. Ten repeats of five-fold cross-validation tests were used to evaluate the anti-HCC drug target gene prediction performances on this DADA dataset. Based on a pre-defined seed gene set, the DADA algorithm would return a ranked gene list as the result. In total, seven types of seed genes, including known anti-HCC drug targets, five types of PDRGs and other genes. After ranking the genes with DADA algorithm, the DADA score of each gene could be calculated by the following equation:

$${\rm{Score}}=\frac{|{candidates}|-{rank}}{|{candidates}|}$$

Where $|{candidates}|$ represents the size of candidate set (i.e. the total number of genes in the DADA dataset), and *rank* represents the rank of each genes in the results of DADA algorithm using one particular set of seed genes. Finally, seven DADA scores were introduced as the input features of one-class SVM model to assess the prediction performance.

### Performance evaluation

Four common metrics were used to illustrate the overall performance of drug target prediction more intuitively. They were sensitivity (Sn), specificity (Sp), Matthew’s correlation coefficient (MCC) and Area Under the Curve (AUC). Sn, Sp and MCC are defined as following equations:

$${\rm{Sn}}=\frac{TP}{TP+FN}$$

$${\rm{Sp}}=\frac{TN}{TN+FP}$$

$${\rm{MCC}}=\frac{(TP\times TN)-(FN\times FP)}{\sqrt{(TP+FN)\times (TN+FP)\times (TP+FP)\times (TN+FN)}}$$

where TP, TN, FP and FN represent true positive, true negative, false positive and false negative, respectively.

To evaluate prediction performance with an independent dataset, we first downloaded protein-drug relationships from DrugBank database. Since DrugBank database doesn’t provide downloadable files including the associated conditions and clinical trial information of drugs, the HTML files were parsed directly to gain these data. Drugs whose associated conditions or clinical information referred to carcinomas were considered as the anti-cancer drugs. As the result, all the genes which were targeted by these anti-cancer drugs were taken as the positive anti-cancer drug target genes in the independent dataset and other genes in the network were used as the negative samples.

## Supplementary information

Identifying potential drug targets in hepatocellular carcinoma based on network analysis and one-class support vector machine

Dataset 1

## References

[1] Ferlay J (2015). Cancer incidence and mortality worldwide: sources, methods and major patterns in GLOBOCAN 2012. International journal of cancer.

[2] Llovet JM, Villanueva A, Lachenmayer A, Finn RS (2015). Advances in targeted therapies for hepatocellular carcinoma in the genomic era. Nature Reviews. Clinical Oncology.

[3] Llovet JM (2016). Hepatocellular carcinoma. Nature Reviews Disease Primers.

[4] Llovet JM, Montal R, Sia D, Finn RS (2018). Molecular therapies and precision medicine for hepatocellular carcinoma. Nature Reviews. Clinical Oncology.

[5] Ringelhan M, Pfister D, O’Connor T, Pikarsky E, Heikenwalder M (2018). The immunology of hepatocellular carcinoma. Nature Immunology.

[6] Forner A, Reig M, Bruix J (2018). Hepatocellular carcinoma. Lancet (London, England).

[7] Plenge RM (2016). Disciplined approach to drug discovery and early development. Science translational medicine.

[8] Arrowsmith J, Miller P (2013). Trial watch: phase II and phase III attrition rates 2011–2012. Nature reviews. Drug discovery.

[9] Hopkins AL (2007). Network pharmacology. Nature biotechnology.

[10] Nault JC, Galle PR, Marquardt JU (2018). The role of molecular enrichment on future therapies in hepatocellular carcinoma. Journal of hepatology.

[11] Mardinoglu A, Boren J, Smith U, Uhlen M, Nielsen J (2018). Systems biology in hepatology: approaches and applications. Nature Reviews Gastroenterology &. Hepatology.

[12] Zhuang L (2014). A network biology approach to discover the molecular biomarker associated with hepatocellular carcinoma. BioMed research international.

[13] Won JK (2017). Protein disulfide isomerase inhibition synergistically enhances the efficacy of sorafenib for hepatocellular carcinoma. Hepatology (Baltimore, Md.).

[14] Lavi O, Skinner J, Gottesman MM (2014). Network features suggest new hepatocellular carcinoma treatment strategies. BMC systems biology.

[15] Zaman N (2013). Signaling network assessment of mutations and copy number variations predict breast cancer subtype-specific drug targets. Cell reports.

[16] Meyers RM (2017). Computational correction of copy number effect improves specificity of CRISPR-Cas9 essentiality screens in cancer cells. Nature genetics.

[17] Tsherniak A (2017). Defining a Cancer Dependency Map. Cell.

[18] Wang J, Li ZX, Qiu CX, Wang D, Cui QH (2012). The relationship between rational drug design and drug side effects. Briefings in bioinformatics.

[19] Hwang WC, Zhang A, Ramanathan M (2008). Identification of information flow-modulating drug targets: a novel bridging paradigm for drug discovery. Clinical pharmacology and therapeutics.

[20] Heikamp K, Bajorath J (2014). Support vector machines for drug discovery. Expert opinion on drug discovery.

[21] Ferrero E, Dunham I, Sanseau P (2017). In silico prediction of novel therapeutic targets using gene-disease association data. Journal of translational medicine.

[22] Bai Li-Yue, Dai Hao, Xu Qin, Junaid Muhammad, Peng Shao-Liang, Zhu Xiaolei, Xiong Yi, Wei Dong-Qing (2018). Prediction of Effective Drug Combinations by an Improved Naïve Bayesian Algorithm. International Journal of Molecular Sciences.

[23] Guo S (2018). Identification and analysis of the human sex-biased genes. Briefings in bioinformatics.

[24] Erten S, Bebek G, Ewing RM, Koyuturk M (2011). DADA: Degree-Aware Algorithms for Network-Based Disease Gene Prioritization. BioData mining.

[25] Bailey MH (2018). Comprehensive Characterization of Cancer Driver Genes and Mutations. Cell.

[26] Wishart DS (2018). DrugBank 5.0: a major update to the DrugBank database for 2018. Nucleic acids research.

[27] Pinero J (2017). DisGeNET: a comprehensive platform integrating information on human disease-associated genes and variants. Nucleic acids research.

[28] Uhlen Mathias, Zhang Cheng, Lee Sunjae, Sjöstedt Evelina, Fagerberg Linn, Bidkhori Gholamreza, Benfeitas Rui, Arif Muhammad, Liu Zhengtao, Edfors Fredrik, Sanli Kemal, von Feilitzen Kalle, Oksvold Per, Lundberg Emma, Hober Sophia, Nilsson Peter, Mattsson Johanna, Schwenk Jochen M., Brunnström Hans, Glimelius Bengt, Sjöblom Tobias, Edqvist Per-Henrik, Djureinovic Dijana, Micke Patrick, Lindskog Cecilia, Mardinoglu Adil, Ponten Fredrik (2017). A pathology atlas of the human cancer transcriptome. Science.

[29] Chatr-Aryamontri A (2017). The BioGRID interaction database: 2017 update. Nucleic acids research.

[30] Zhou Y, Cui Q (2018). Comparative Analysis of Human Genes Frequently and Occasionally Regulated by m(6)A Modification. Genomics, proteomics &. bioinformatics.

[31] Hagberg, A., Swart, P., Chult, S. & Exploring, D. Network structure, dynamics, and function using networkx, https://www.osti.gov/servlets/purl/960616 (2008).

[32] Shannon P (2003). Cytoscape: a software environment for integrated models of biomolecular interaction networks. Genome research.

[33] Chang C-C, Lin C-J (2011). Libsvm: A Library for Support Vector Machines. ACM Transactions on Intelligent Systems and Technology.

