# Supplementary material for: Identifying potential drug targets in hepatocellular carcinoma based on network analysis and one-class support vector machine
Source: Sci Rep. 2019 Jul 18;9:10442. doi: 10.1038/s41598-019-46540-x (PMC6639372; doi:10.1038/s41598-019-46540-x)
Supplement: Supplementary file 1 — Identifying potential drug targets in hepatocellular carcinoma based on network analysis and one-class support vector machine [file 41598_2019_46540_MOESM1_ESM.docx]

**SUPPLEMENTARY MATERIALS**

**Identifying potential drug targets in hepatocellular carcinoma based on network analysis and one-class support vector machine**

Zhan Tong^a^, Yuan Zhou^a,*^, Juan Wang^a,*^

^a^Department of Biomedical Informatics, School of Basic Medical Sciences, Peking University, Beijing 100191, China

* To whom correspondence should be addressed: Juan Wang (E-mail: [wjuan@hsc.pku.edu.cn](mailto:wjuan@hsc.pku.edu.cn)). Correspondence may also be addressed to Yuan Zhou (E-mail: [zhouyuanbioinfo@hsc.pku.edu.cn](mailto:zhouyuanbioinfo@hsc.pku.edu.cn))

E-mail for other co-authors: Zhan Tong ([tongzhan@hsc.pku.edu.cn](mailto:tongzhan@hsc.pku.edu.cn))

**SUPPLEMENTARY TABLES**

**Supplementary Table S1.** Performance summary of ten repeats of five-fold cross-validation tests of the one-class SVM predictor based after excluding all network centrality features.

| Sensitivity | Specificity | MCC | AUC |
| --- | --- | --- | --- |
| 60.37% | 91.22% | 0.1967 | 0.8834 |
| 43.78% | 94.87% | 0.1870 | 0.8824 |
| 60.83% | 90.96% | 0.1950 | 0.8840 |
| 59.45% | 90.86% | 0.1888 | 0.8834 |
| 57.60% | 91.57% | 0.1910 | 0.8849 |
| 49.31% | 93.59% | 0.1880 | 0.8830 |
| 62.21% | 90.26% | 0.1916 | 0.8845 |
| 60.83% | 90.49% | 0.1893 | 0.8840 |
| 63.13% | 89.77% | 0.1891 | 0.8828 |
| 60.83% | 90.55% | 0.1901 | 0.8855 |

**Supplementary Table S2.** Performance summary of ten repeats of five-fold cross-validation tests of the one-class SVM predictor after including additional network centrality features, including PageRank centrality, eigenvector centrality and Katz centrality

| Sensitivity | Specificity | MCC | AUC |
| --- | --- | --- | --- |
| 61.75% | 90.90% | 0.1977 | 0.8841 |
| 66.82% | 88.38% | 0.1872 | 0.8833 |
| 61.75% | 90.40% | 0.1915 | 0.8834 |
| 59.91% | 90.50% | 0.1862 | 0.8833 |
| 53.00% | 92.71% | 0.1892 | 0.8843 |
| 49.77% | 93.34% | 0.1858 | 0.8823 |
| 61.75% | 90.53% | 0.1931 | 0.8852 |
| 58.99% | 90.89% | 0.1875 | 0.8848 |
| 65.90% | 88.98% | 0.1902 | 0.8838 |
| 64.98% | 88.77% | 0.1850 | 0.8836 |

**Supplementary Table S3.** Comparison of prediction models using primary features and DADA score.

| Feature set | Sensitivity | Specificity | MCC | AUC |
| --- | --- | --- | --- | --- |
| Primary features | 0.4212 (±0.0564) | 0.9398 (±0.0147) | 0.1651 (±0.0032) | 0.8569 (±0.0045) |
| DADA score | 0.3648 (±0.1303) | 0.9553 (±0.0263) | 0.1773 (±0.0043) | 0.8162 (±0.0077) |
| Replacing network centrality features with DADA score | 0.3896  (±0.0343) | 0.9497 (±0.0083) | 0.1673 (±0.0027) | 0.8558 (±0.0040) |
| Primary features + DADA score | 0.3870 (±0.0325) | 0.9499 (±0.0078) | 0.1664 (±0.0034) | 0.8562 (±0.0041) |

**Supplementary Table S4.** The features used by the one-class SVM predictor

| Category | Feature name | Total |
| --- | --- | --- |
| Network centrality features | Degree_centrality, Betweenness_centrality, Closeness_centrality | 3 |
| Network distance features | DAG_mean_dist, DAG_min_dist, DRG_mean_dist, DRG_min_dist, URG_mean_dist, URG_min_dist, others_mean_dist, others_min_dist, PFG_mean_dist, PFG_min_dist, PUG_mean_dist, PUG_min_dist, DTG_mean_dist, DTG_min_dist | 14 |
| Network neighborhood features | DAG_ratio, PFG_ratio, PUG_ratio, DRG_ratio, URG_ratio, others_ratio, DTG_ratio | 7 |
| Genetic dependency score features | HLF_LIVER, HUH1_LIVER, HUH6_LIVER, HUH7_LIVER, JHH1_LIVER, JHH4_LIVER, JHH5_LIVER, JHH7_LIVER, LI7_LIVER, PLCPRF5_LIVER, SKHEP1_LIVER, SNU182_LIVER, SNU398_LIVER, SNU449_LIVER | 14 |
| Genetic dependency score features of its network neighbors | HLF_LIVER_nei, HUH1_LIVER_nei, HUH6_LIVER_nei, HUH7_LIVER_nei, JHH1_LIVER_nei, JHH4_LIVER_nei, JHH5_LIVER_nei, JHH7_LIVER_nei, LI7_LIVER_nei, PLCPRF5_LIVER_nei, SKHEP1_LIVER_nei, SNU182_LIVER_nei, SNU398_LIVER_nei, SNU449_LIVER_nei | 14 |

Six types of annotated genes are considered here, including disease-associated genes (DAGs), prognostic favorable genes (PFGs), prognostic unfavorable genes (PUGs), up-regulated genes (URGs) and down-regulated genes (DRGs) and drug target genes (DTGs). Min_dist, minimum distance to one type of annotated genes, mean_dist, mean distance to one type of annotated genes, nei, neighbor genes.

**SUPPLEMENTARY FIGURES**

**
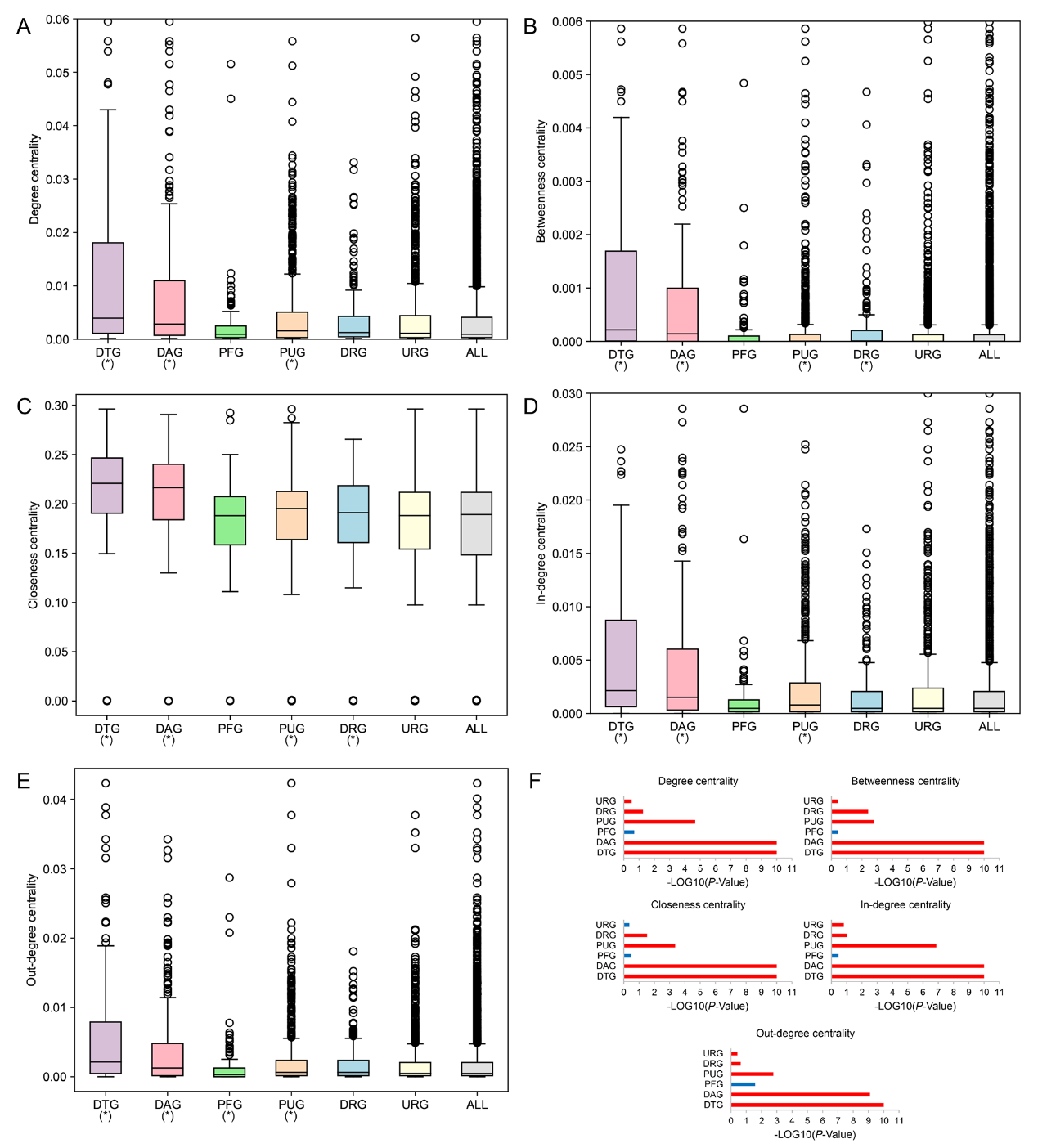
**

**Supplementary Figure S1. Network centrality characteristics of different types of genes in human cellular signaling network.** (A) Degree centrality. (B) Betweenness centrality. (C) Closeness centrality. (D) In-degree centrality. (E) Out-degree centrality. *P < 0.05 from Wilcoxon test. (F) P-values of comparing centralities of DTGs and different types of PDRGs with those of background. Red and blue bars represent the greater and less centralities compared to the background, respectively.


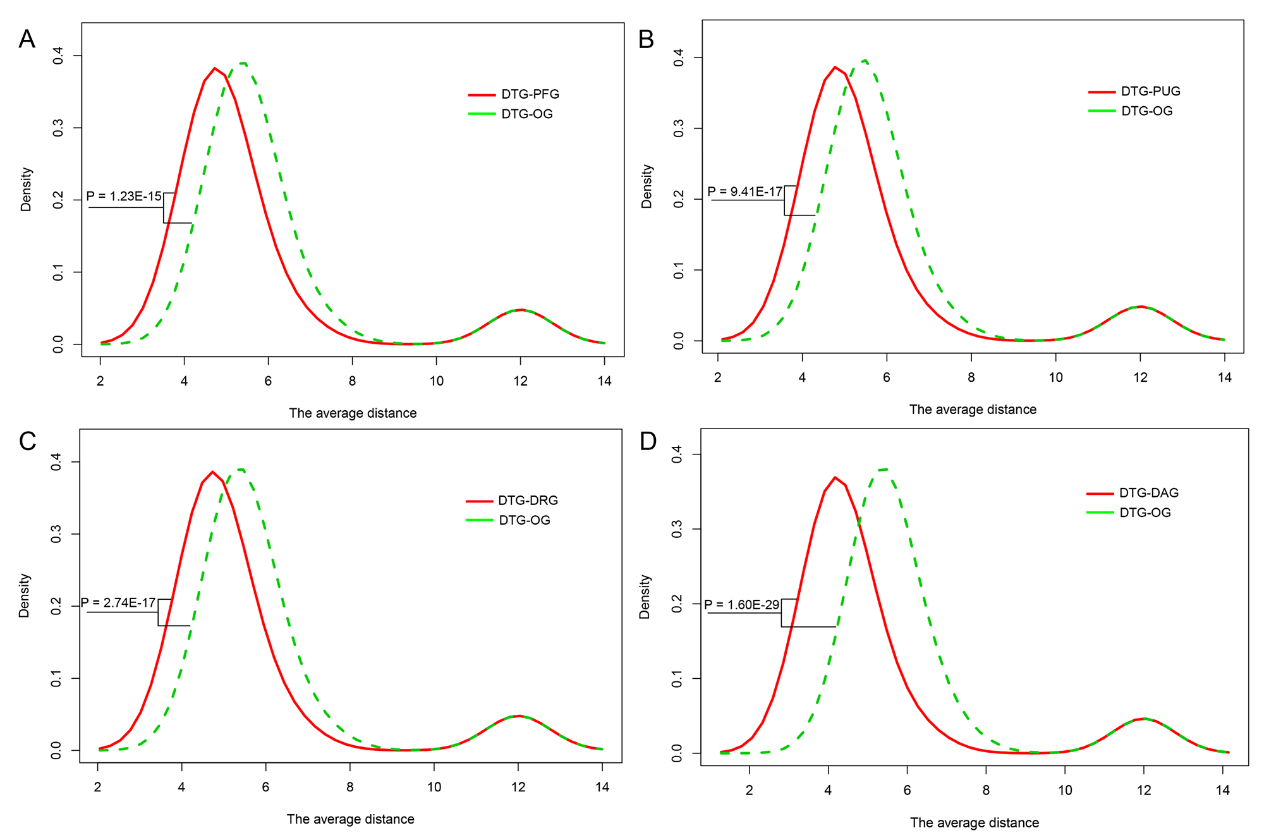


**Supplementary Figure S2. Network distance characteristics between DTGs and the specific PDRGs in human cellular signaling network.** Density plots display the probability distribution of the average lengths of shortest paths between groups (DTG-PDRG/DTG-other genes).


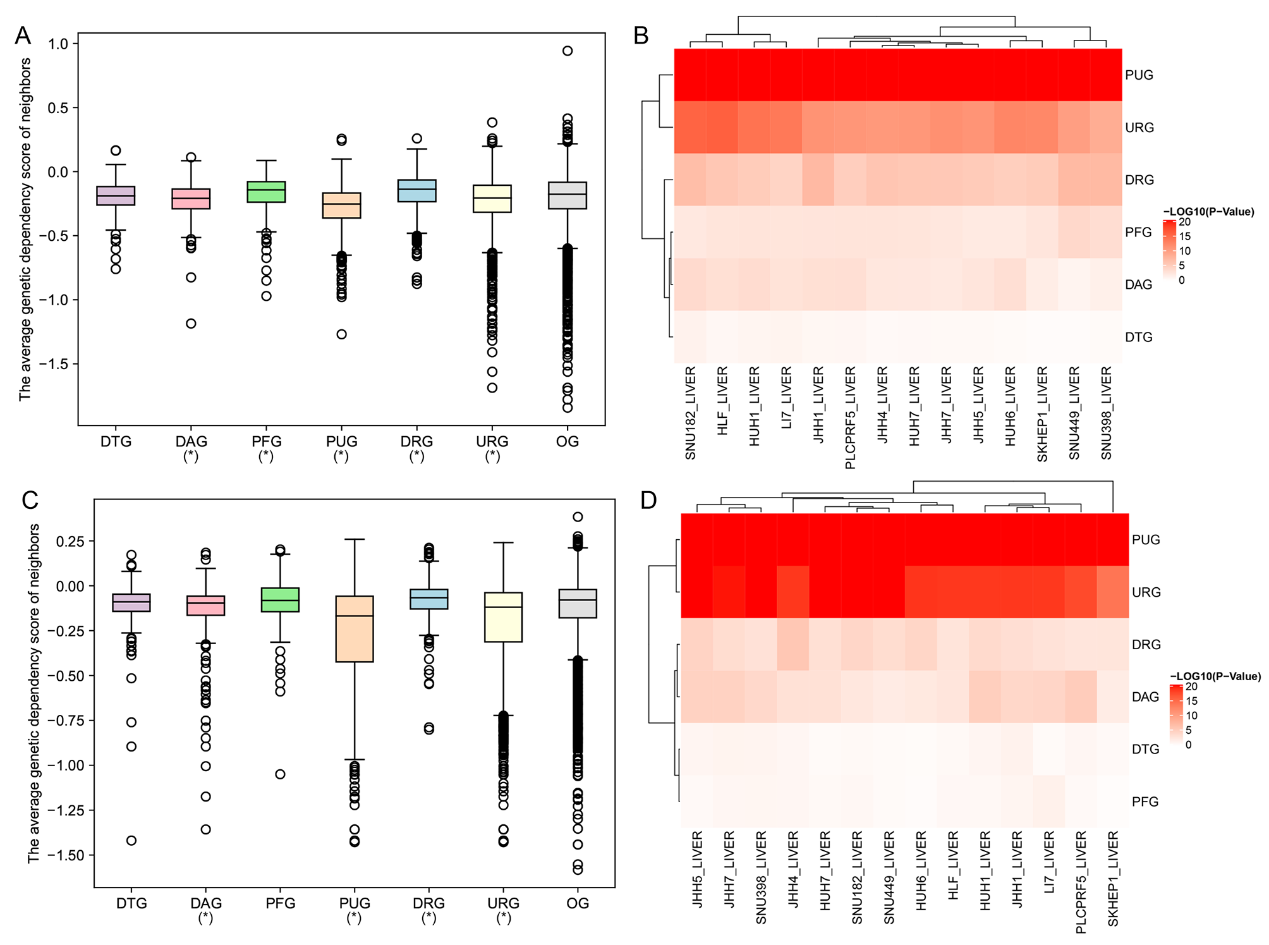


**Supplementary Figure S3. Genetic dependency score characteristics of different types of genes’ neighbors in human PPI network and the signaling network.** Box plots display the average genetic dependency score differences among neighbors of different gene groups and background in human PPI network (A) and the signaling network (C). *P < 0.05 from Wilcoxon test. Heat-maps display the p-values of comparing the average genetic dependency scores of different types of genes’ neighbors with the background in human PPI network (B) and the signaling network (D) with Wilcoxon test in 14 HCC cell-lines.

**
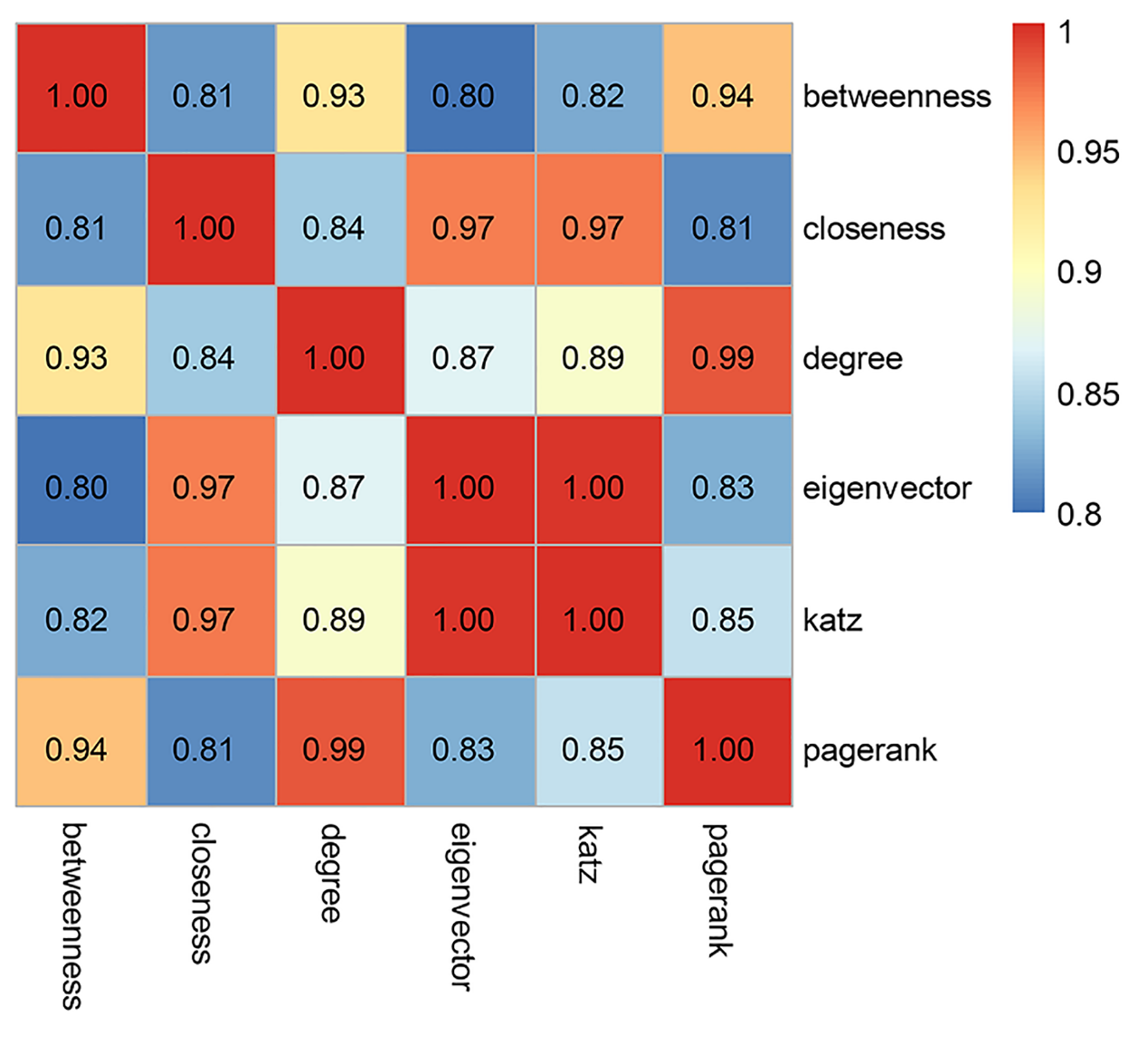
**

**Supplementary Figure S4. Correlation characteristics of each pair of centrality metrics.** Heat-map displays the spearman correlation coefficient for each pair of centrality metrics.
